# Asymptomatic malaria infection, associated factors and accuracy of diagnostic tests in a historically high transmission setting in Northern Uganda

**DOI:** 10.1186/s12936-022-04421-1

**Published:** 2022-12-23

**Authors:** Bosco B. Agaba, Simon P. Rugera, Ruth Mpirirwe, Martha Atekat, Samuel Okubal, Khalid Masereka, Miseal Erionu, Bosco Adranya, Gertrude Nabirwa, Patrick B. Odong, Yasin Mukiibi, Isaac Ssewanyana, Susan Nabadda, Enoch Muwanguzi

**Affiliations:** 1 Department of Medical Laboratory Science, Mbarara University of Science and Technology, Mbarara, Uganda; 2 National Malaria Control Division, Ministry of Health, Kampala, Uganda; 3 Department of Statistics, Makerere University, Kampala, Uganda; 4 Uganda Institute of Allied and Management Sciences, Kampala, Uganda; 5 National Malaria Reference Laboratory, Central Public Health Laboratory Services, Kampala, Uganda; 6 Infectious Diseases Research Collaboration, Kampala, Uganda

**Keywords:** Asymptomatic malaria infections, Rapid diagnostic, Blood smear microscopy

## Abstract

**Background:**

Asymptomatic malaria infections are important parasite reservoirs and could sustain transmission in the population, but they are often unreported. A community-based survey was conducted to investigate the prevalence and factors associated with asymptomatic malaria infections in a historically high transmission setting in northern Uganda.

**Methods:**

Using a cross-sectional design, 288 children aged 2–15 years were enrolled and tested for the presence of malaria parasites using rapid diagnostic tests (RDTs) and blood smear microscopy between January to May 2022. Statistical analysis was performed using the exact binomial and Fisher’s exact test with p ≤ 0.05 indicating significance. The logistic regression was used to explore factors associated with asymptomatic malaria infections.

**Results:**

Overall, the prevalence of asymptomatic infection was 34.7% (95% CI 29.2–40.5) with the highest observed in children 5–10 years 45.9% (95% CI 35.0–57.0). Gweri village accounted for 39.1% (95% CI 27.6—51.6) of malaria infections. Median parasite density was 1500 parasites/µl of blood. *Plasmodium falciparum* was the dominant species (86%) followed by *Plasmodium malariae* (5%). Factors associated with asymptomatic malaria infection were sleeping under mosquito net (Adjusted Odds Ratio (aOR) 0.27; 95% CI 0.13–0.56), p = 0.001 and presence of village health teams (VHTs) (aOR 0.02; 95% CI 0.01–0.45), p = 0.001. Sensitivity and specificity were higher for the *P. falciparum*/pLDH RDTs compared to HRP2-only RDTs, 90% (95% CI 86.5–93.5) and 95.2% (95% CI 92.8–97.7), p = 0.001, respectively.

**Conclusion:**

Asymptomatic malaria infections were present in the study population and this varied with place and person in the different age groups. *Plasmodium falciparum* was the dominant parasite species however the presence of *P. malariae* and *Plasmodium ovale* was observed, which may have implication for the choice and deployment of diagnostic tools. Individuals who slept under mosquito net or had presence of functional VHTs were less likely to have asymptomatic malaria infection. P.f/pLDH RDTs performed better than the routinely used HRP2 RDTs. In view of these findings, investigation and reporting of asymptomatic malaria reservoirs through community surveys is recommended for accurate disease burden estimate and better targeting of control.

**Supplementary Information:**

The online version contains supplementary material available at 10.1186/s12936-022-04421-1.

## Background

The WHO African Region continues to contribute a disproportionately high share of the global malaria burden accounting for 95% of malaria cases [1]. Uganda is categorized among the six highest malaria burden countries [2] and malaria remains a major public health problem in the country causing 16 million cases annually [3–5]. Although there is variation in the epidemiology of malaria in Uganda, the whole country is endemic and transmission occurs throughout the year. *Plasmodium falciparum* accounts for > 95% of malaria infections in Uganda [5–7]. Asymptomatic malaria infections are often undetected, not reported and remain in the communities contributing to transmission [8, 9].

In Uganda, several studies have reported a high burden of asymptomatic infections in children [10]. Mathematical and epidemiological modeling has shown the importance of addressing asymptomatic infections and their potential to derail malaria elimination efforts [11]. Aduku located in Northern Uganda is traditionally an epicentre for malaria transmission reporting one of the highest entomological inoculation rates (EIR) in the World with approximately 1500 infective bites per year [14–16]. The area is one of those that received indoor residual spraying (IRS) in addition to the use of long-lasting insecticidal nets (LLINs), intermittent preventive therapy (IPT) and diagnosis and treatment of cases for malaria control [6, 12]. Despite the control interventions, previous studies have highlighted the potential of asymptomatic infections to sustain transmission that impacts on malaria elimination efforts [13]. While routine health management information system (HMIS) data shows a marked reduction in malaria cases in this area, the trend and pattern of asymptomatic malaria infection in the communities are unclear.

As part of the efforts to control malaria in this region, the Ministry of Health established a sentinel-surveillance site in Aduku to conduct therapeutic efficacy studies and collect high-quality malaria data to inform control interventions. The HMIS and the mid-term review of the 2015 malaria strategic plan have reported reductions in malaria burden in Aduku, however, these estimates are majorly based on symptomatic individuals, who come for care and treatment at health facilities [3].

In addition to symptomatic cases, the country’s malaria control policy recommends the identification and clearance of parasites in asymptomatic infections as an important intervention for malaria elimination. However, there is no recent data on the burden of asymptomatic parasite reservoirs in communities living in Aduku and burden remains unknown. Asymptomatic infections are important malaria parasite reservoirs which sustain malaria transmission in communities [17] that compromises and threatens malaria elimination efforts [18]. Lack of data on asymptomatic malaria parasite reservoir potentially under-estimates burden, undermines efforts for parasite clearance and compromise opportunities for transmission interruption and subsequent efforts to achieve malaria elimination. The study aim was to investigate the prevalence and factors associated with asymptomatic malaria infections in Aduku.

## Methods

### Study design

This was a household community-based cross-sectional study that enrolled participants from a random sample of villages and households in Aduku sub-county. The first stage of sampling was at sub-county level where a list of all parishes in the sub-county was obtained which formed the first sampling frame. Using simple random sampling, four (4) parishes of Apire, Alira, Aboko and Ongoceng were selected. The second stage of sampling was done at the parish where a list of all villages in each of the 4 parishes formed a sampling frame. From each parish, one village was randomly selected. The selected villages were Gweri, Egum, Akwon and Amia A. From each village, a random sample of households was selected from which eligible children were enrolled into the study.

### Study area and setting

**Fig. 1:**
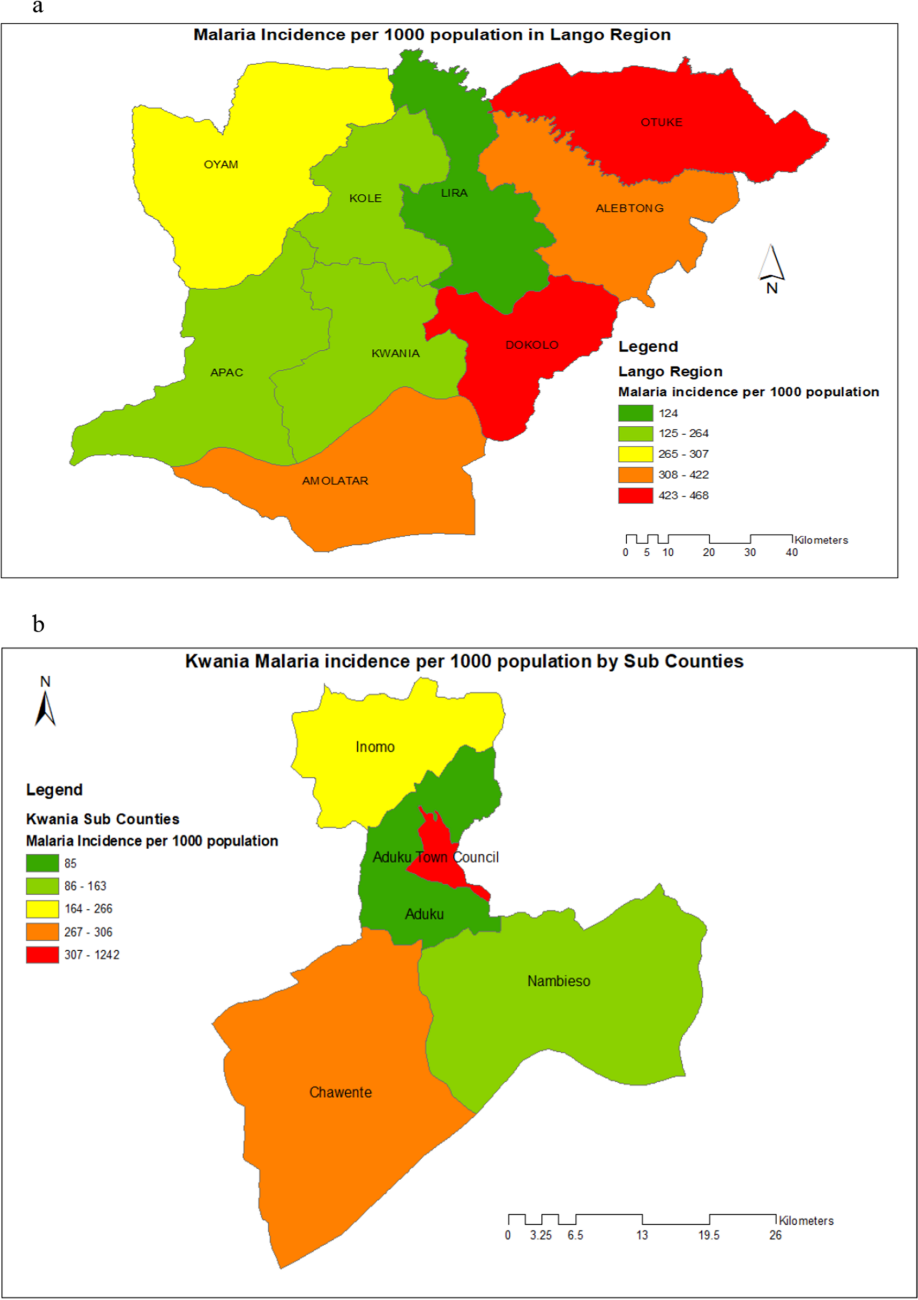
Geographical information system (GIS) mapping of the study areas. **1a**: Geographical information system (GIS) mapping of Lango region showing the location of Kwania district where Aduku sub-county is located. **1b**: Geographical information system (GIS) mapping of Kwania district showing the location of Aduku Sub- County where the study took place

The study was conducted in four randomly selected villages and parishes in Aduku sub-county in Kwania district. Aduku is located approximately 282 km north of Kampala city. The geographical positioning/coordinates of Aduku are: 2°01′10.0"N, 32°43′12.0"E (Latitude:2.0194; Longitude:32.7200). It is traditionally a high malaria transmission area with one of the highest EIR ever reported in the world because of the conducive climatic and environmental conditions optimal for breeding of malaria vectors [14–16]. Aduku is one of the malaria sentinel surveillance sites operated by the Ministry of Health that reports high quality data and has strong laboratory capacity. The study area is shown in Fig. 1a and b.

### Study population

Children aged 2–15 years living in the selected parishes and villages in the selected households in Aduku sub-county were enrolled into the study if they met the eligibility criteria. Where children were unable to assent, the care taker provided the required information. Children’s care takers or guardians were administered a study questionnaire to collect additional demographic variables. The rationale for choosing the 2–15 years age group as the study population was based on evidence from recent studies that showed rapid shifts in parasitaemia with higher parasite prevalence seen in older children above 5 years as compared to children under 5 [19, 20].

### Sample size determination

The study sample size was determined using the Kish and Leslie (1965) formula for cross-sectional surveys (WHO, 2011) based on the following assumptions. The expected proportion of 13% was assumed for asymptomatic malaria infections in this region based on the Uganda National indicator survey [4]. A standard Z-score (1.96) and a precision or allowable margin of error of (0.05) were used. A design effect of 1.5 was factored in to cater for multi-stage sampling and a 10% for non-response rate to determine the minimum sample required for this study.

### Eligibility criteria

The inclusion criteria were children aged 2–15 years with an auxiliary temperature of < 37.5 °C who provided assent and whose guardian or caretakers allowed them to participate and provided a blood sample for malaria parasites. Exclusion criteria was presence of signs and symptoms of severe malaria (these were immediately referred to the facility) and those who were presently on treatment for malaria.

### Data collection and laboratory procedures

Data collection was done using a short questionnaire which captured individuals’ details and demographics from consented participants. Permission to enroll eligible children was obtained from the care taker or older children who were able to assent. Each enrolled child provided approximately two drops of blood obtained by finger prick for malaria testing. Malaria testing was done by two different malaria RDTs (SD Bioline, Cat no. 05FK50 and 05FK90, S. Korea) and blood smear microscopy for each participant. Results of the RDTs were provided to study participants immediately on site while blood smears were collected, dried and transported to Aduku HC IV malaria sentinel surveillance site for staining and microscopy. Both thick and thin smears were collected on same slide.

### Blood smear microscopy

Blood smears were prepared in the field using blood samples obtained from a finger- prick with both thick and thin smears on same slide. Thick smears were used for parasite detection and quantification while thin smear for species identification. Thin smear was fixed with absolute methanol and thereafter the slides kept in the field in a slide box for no longer than 12 h to avoid auto-fixation. At Aduku sentinel site field laboratory, thick blood smears were stained with 10% Giemsa for 30 min before transportation to the malaria research laboratory in Kampala for examination for the presence of parasitaemia. All slides were cross-checked and re-examined by the WHO certified expert slide readers at the research laboratory. Parasite densities were calculated from thick blood smears by counting the number of asexual parasites and reported as parasites per micro litre of blood. A thick blood smear was considered negative when the examination of 100 high power fields did not reveal asexual parasites. For quality control, all slides were read by two independent microscopists and a third reviewer settled any discrepant readings. The standard WHO procedure for Giemsa staining and reading of blood smears was strictly followed [21].

### Rapid diagnostic tests (RDTs)

RDTs were done according to the manufacturer’s instructions. Two malaria RDTs P.f-HRP2/(pLDH) Cat. 05FK90 and P.f-HRP2 Cat. 05FK50 (SD Bioline, S. South Korea) were used in the study. Briefly, a drop of blood was placed in the sample well, followed by dropping the buffer into the buffer well. The buffer lyses the RBCs exposing the target antigen and enhance the flow of the sample along the nitrocellulose strip. Appearance of the test and control indicates presence of parasites while presence of control without test line means absence of parasite antigens. The RDT test procedure were done according to the manufacturer’s recommendations.

### Ethical considerations

The study was approved by the Mbarara University Research and Ethics Committee. Additional approval to conduct the study was obtained from the Kwania district health office. All participants provided written consent before enrollment into the study.

### Study profile

**Fig. 2:**
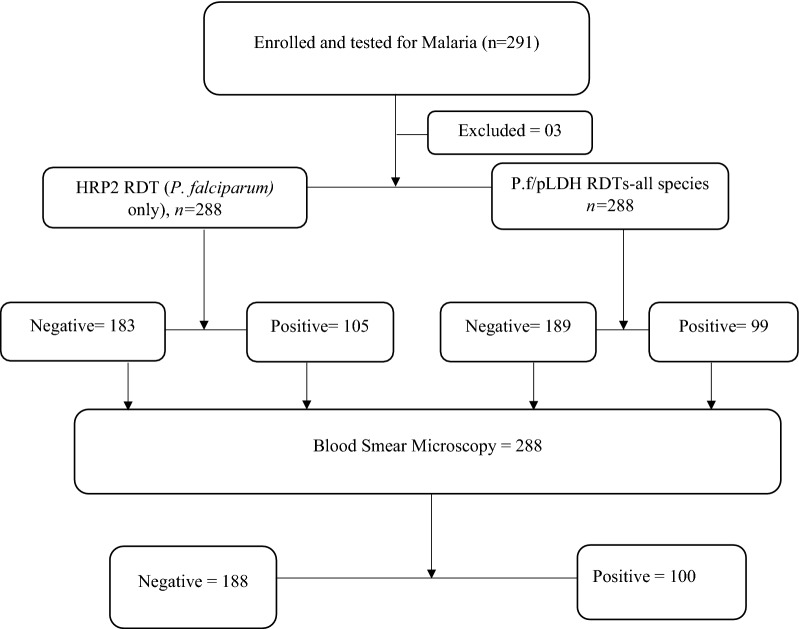
Detailed process of participant enrolment

A total of 291 participants were enrolled into the study. Three (3) samples were excluded leaving 288 for analysis and reporting. The detailed enrolment and study flow chart is illustrated in Fig. 2.

## Results

### Population characteristics and demographics

The study estimated the proportion of asymptomatic malaria in children 2–15 years in Aduku sub-county in Kwania district. Overall, 52.8% of the enrolled participants were female. The majority of the participants were in the 10–15 years age bracket. Although Amia-A village had the biggest number of participants, the sample was evenly distributed across the study villages. The detailed baseline characteristics are indicated in Table 1.

**Table 1:** Baseline characteristics of the study participants

Variable	Frequency	Proportion (%)
Sex		
Male	136	52.8
Female	152	47.2
Age		
< 5	82	28.5
5 to 10	85	29.5
10.1 to 15	121	42
Village		
Akwon	70	24.3
Amia A	79	27.4
Egum	70	24.3
Gweri	69	23.9
Fever in past 2 weeks		
No	98	34.4
Yes	187	65.6
Care taker's Education		
Primary	15	8.1
Secondary	165	89.2
Tertiary	5	2.7
Knowledge on Cause of malaria		
Mosquito	274	97.2
Parasite	7	2.5
Others	1	0.35
Recognition of symptoms		
Yes	256	91.1
No	25	8.9
Malaria can be treated		
Yes	279	99.3
No	2	0.71
Mosquito Biting times		
At Night	270	96.1
Day Time	7	2.5
Not Sure	4	1.4
Breeding sites		
Bushes	5	1.8
Stagnant water	275	97.9
Not sure	1	0.4
Mosquito Net ownership		
Yes	224	79.2
No	59	20.9
Slept under a Net previous Night		
No	116	41.1
Yes	166	58.8

Summary of baseline characteristics

### Prevalence of asymptomatic malaria

Overall, 34.7% (95% CI 29.2–40.5) of the participants had asymptomatic malaria infection by blood smear microscopy which is significantly higher than the regional parasite prevalence (13%) for Lango region [4]. The asymptomatic infections were higher in children between 5 and 10 years of age (45.9%, 95% CI 35.0–57.0). Gweri village carried the highest burden of malaria infections (39.1%, 95% CI 27.6–51.6) Table 2.

**Table 2:** Prevalence of asymptomatic malaria infections

Variable	Prevalence of asymptomatic malaria by blood smear microscopy	
	Proportion (%)	95% CI
Overall prevalence	34.7	(29.2–40.5)
Prevalence by age		
< 5 year	29.3	(19.7–40.4)
5 to 10 years	45.9	(35.0–57.0)
10.1 to 15 years	30.6	(22.5–39.6)
Prevalence by village		
Akwon	31.4	(20.8–43.6)
Egum	32.9	(22.1–45.1)
Gweri	39.1	(27.6–51.6)
Amia A	35.4	(25.0–47.0)

### *Plasmodium* parasites species among asymptomatic infections

A majority of malaria infections were due to *P. falciparum* (86.0%). However other malaria species were encountered in this study population, *Plasmodium malariae* (5%), and *Plasmodium ovale* (1%) Table 3.

**Table 3:** Parasites species composition in asymptomatic malaria infections

Village Name	Parasite species by blood smear microscopy among asymptomatic malaria infections					Total
	*P. falciparum*	*P. malariae*	*P. ovale*	Mixed infection (Pf, Pm	Mixed infection (Pf, Po)	
Akwon	21	0	0	1	0	22
Egum	22	2	1	2	1	28
Gweri	21	1	0	1	0	23
Amia A	22	2	0	3	0	27
Total	86	5	1	7	1	100

### Parasite density by age and village

**Fig. 3:**
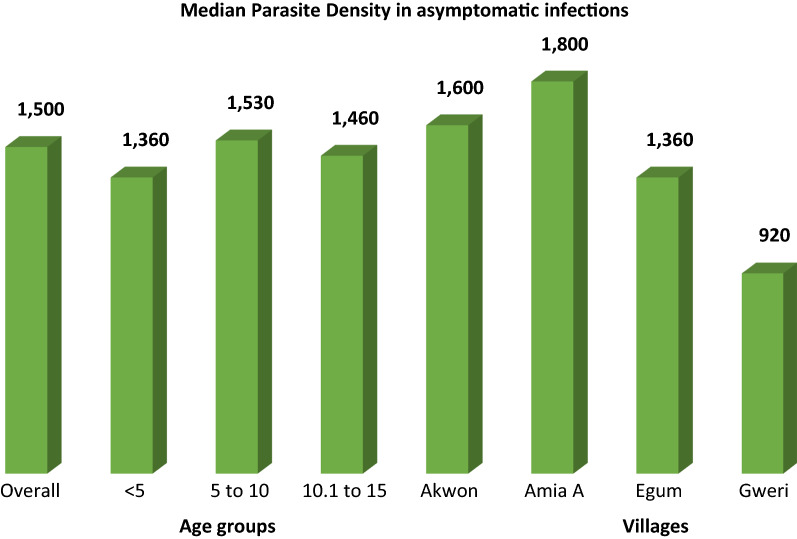
Median parasite density in Aduku sub-county

Overall, the median parasite load in the study population was 1500 parasites per microlitre of blood. The highest parasite density was observed in children of 5–10 years of age (median, 1530 parasites/microlitre). Most of the high-density infections were seen in Amia A village (median density 1800) (Fig. 3).

### Factors associated with asymptomatic malaria infection

The possible factors associated with asymptomatic malaria infection were assessed using a logistic regression model with odds ratio (OR) as measure of association. Bivariable and multivariable analysis were done to obtain the crude and adjusted estimates respectively. The factors associated with asymptomatic malaria infection were presence of a village health team aOR = 0.02 (95% CI 0.006–0.45), p = 0.001 and sleeping under a mosquito net the previous night aOR = 0.269 (0.130–0.557), p = 0.001 (Table 4).

**Table 4:** Factors associated with asymptomatic malaria infection in the study population

Variable	OR (95% CI)	p- value	aOR (95% CI)	p-value
Age				
< 5	Reference			
5.0 to 10	0.46 (0.24, 0.87)	0.017	0.50 (0.20, 1.30)	0.155
10.1 to 15	0.91(0.49, 1.68)	0.767	0.80 (0.31, 2.06)	0.647
Sex				
Female	Reference			
Male	1.01(0.62, 1.65)	0.956	0.65 (0.31, 1.35)	0.248
Village				
Akwon	Reference			
AmiaA	0.84 (0.42, 1.65)	0.856	0.74 (0.27, 2.03)	0.564
Egum	0.94 (0.46, 1.90)	0.343	1.23 (0.42,3.61)	0.705
Gweri	0.71 (0.35, 1.43)	0.605	1.08 (0.35, 3.29)	0.899
Recognition of Malaria symptoms				
Fever	Reference			
Others	1.19 (0.54,2.62)	0.662	0.59 (0.21, 1.69)	0.328
Presence of a VHT of CHW				
No	Reference			
Yes	0.15 (0.01, 0.41)	0.001	0.02 (0.01, 0.45)	0.001
Slept under net previous night				
No	Reference			
Yes	0.22 (0.13, 0.38)	0.001	0.27 (0.13,0.56)	0.001

### Field performance of the different diagnostic tests used in the study

During the study, two different RDTs were used for malaria in addition to blood smear microscopy. The sensitivity and specificities of the different tests used in the study were evaluated to assess their field performance in this population using expert blood smear as the gold standard. Overall, the P.f/pLDH RDTs were better for both sensitivity and specificity 90% (86.5–93.5) and 95.2% (92.8–97.7), p = 0.001, respectively. The predictive values followed the same trend and were better for the P.f/pLDH RDTs, 90.9% (87.6–94.2) and 94.7% (92.1–97.3), p = 0.001 for PPV and NPV, respectively (Table 5).

**Table 5:** Performance of the different diagnostic tests

Performance of the different tests used in the study compared to blood smear microscopy					
	Pf-HRP2	95% CI	Pf-HRP2/(pLDH)	95% CI	p- value
Sensitivity (%)	85	80.88–89.12	90	86.54–93.46	0.0001
Specificity (%)	89	85.80–92.92	95.21	92.75–97.68	0.0034
PPV	80	76.42–85.49	90.91	87.59–94.23	0.0023
NPV	91	88.64–94.97	94.71	92.12–97.29	0.0001

## Discussion

The study investigated the prevalence of asymptomatic malaria infection in Aduku sub-county Kwania district in Lango region a historically high malaria transmission area [3, 4]. The statistical hypothesis driving the study was that there was no difference in the prevalence of asymptomatic malaria infection in the population living in Aduku compared to the 13.0% population prevalence for Lango region.

### Prevalence of asymptomatic malaria infection

The prevalence of asymptomatic malaria infection was investigated using a community-based surveillance system in four randomly selected parishes and villages in Aduku sub-county. Overall, 34.7% of individuals tested for malaria in this community had parasites. The observed high malaria burden in Aduku is consistent with what was reported previously in Northern Uganda [22–24]. However, it is higher than what was reported in Tanzania [25], Ghana [26], Ethiopia [27] and Myanmar [28], but lower than (81.1%) reported in a community survey in Cameroon [29] and in another study in Ghana [30]. Historically, Aduku has been categorized as a high malaria transmission area reporting one of the highest entomological inoculation rates (EIR) in the world [15, 16, 23]. The factors and drivers for high malaria transmission rates in Aduku have been reported as: a high EIR that drives transmission intensity, waning immunity and suboptimal coverage of intervention [15, 16, 20, 31]. The presence of high proportions of asymptomatic malaria infections in communities has negative implications for malaria control interventions as it provides parasites reservoir that sustains transmission in communities [32].

Prevalence was disproportionately higher in children 5–10 years old 45.9% (95% CI 35.0–57.0) followed by the 10–15 years 30.6% (95% CI 22.5–39.6). Traditionally, children < 5 years carried the highest malaria burden in Uganda due to lower immunity [7], however recent studies have reported a shift in malaria parasite burden from the under -five to older children above 5 years of age [20, 31]. Similar shifts of malaria burden from the < 5 years to the older children has been reported in many studies in eastern and parts of Lango and Acholi in Uganda [20, 31, 33]. Several studies elsewhere have predicted similar higher prevalence of malaria burden among older age groups above 5 years following reduced transmission due to impact of control interventions [31, 34]. The observed shift in parasitaemia from the < 5 to older children has been explained by the waning immunity as well as increased exposure to infective bites in older children. The under-fives have been targeted by the numerous mass campaigns of mosquito nets and may have benefited from the protective effect of increased use of insecticide-treated nets relative to older age groups [20, 31]. Behavioural factors including occupational activities such as agriculture or night-time work may have increased the risk of exposure outside the household for older children as compared to the < 5 years [20].

### Parasite density

Parasite density is a quantitative method that provides an estimate of malaria parasites in an infected individual. While there are several parasite quantification methods including counting parasites/200 WBC, the plus system and quantitative PCR (qPCR), the WHO recommends the use of blood smear microscopy for quantification and reporting of parasite counts per microlitre of blood assuming a standard WBC count of 8,000 cells for healthy individuals [21]. Using expert microscopy, the median parasite density in the study population was 1500 parasites/microlitre of blood. The observed relatively high density is consistent with high malaria transmission setting. Parasite quantification can be an important parameter for malaria case management as it helps in identification and classification of severity of disease [35]. Several studies elsewhere have reported association between hyper-parasitaemia and severe malaria with pathophysiological consequences of disease [36], however other studies have shown conflicting evidence [37]. Higher parasite densities in asymptomatic cases were associated with increased odds of developing symptomatic malaria [36] which further emphasizes the importance of parasite density estimation and treatment of asymptomatic cases. Other studies have suggested a linkage between parasite density and malaria transmission intensity, high density infections being characteristic of high transmission setting [31, 38]. Parasite density can also predict accuracy of malaria diagnostic tools as low-density (LD) *Plasmodium* infections have been reported to be missed by standard malaria rapid diagnostic tests when the blood antigen concentration is below the detection threshold [39].

Epidemiologically in terms of person, time and place; high-density malaria infections (> 1000/µl) were disproportionately seen in children 5–10 and 10–15 years and in Gweri village in this study population. The presence of high-density infection in relatively older children (above 5 years) is consistent with what was reported previously in Uganda and Tanzania and may provide additional evidence of shifts of parasitaemia from the < 5 to older children [31, 38]. The possible explanation for shifts in parasitaemia to older children is related to the waning immunity, occupation and heavy focus of control interventions to the < 5 years of age [20]. Variation in parasite density between villages may be explained by the differences in population characteristics between places, immunity and coverage of intervention.

### Parasite species

Speciation of plasmodium species is an important malaria epidemiological parameter for understanding a country’s parasite population that eventually informs public health control interventions for malaria. In this study, the dominant species was *P. falciparum* (86%) followed by *P. malariae* (5%). *Plasmodium ovale* was present, but in extremely low proportions (1%). Predominance of *P. falciparum* in this study is similar to what is reported in the MOH national malaria survey (MIS) [4]. The observed presence of non*-P. falciparum* species in these samples is also consistent with the results of 2019 MIS that reported an increase in non-*falciparum* species, particularly *P. malariae* and *P. ovale,* in Uganda [4, 40]. Similar studies have previously reported presence of the different parasite species in the same region in Uganda [24, 38, 40] and elsewhere [26, 30, 41, 42]. *Plasmodium falciparum* is the most pathogenic species causing the most aggressive form of malaria (severe malaria) [1, 35]. The implication of its presence in high proportion in asymptomatic individuals in this population is the possible risk of continued and sustained transmission which undermines current control efforts. The presence of other species other than *P. falciparum* has implication on the type of RDTs to be deployed in this setting since the current HRP2 tests only detects *P. falciparum* mono-infection. Non-*P. falciparum* can cause false negative RDTs in settings where *P. falciparum* only RDTs are exclusively used [38]. In addition, the presence of mixed infections of *P. falciparum* + *P. malariae*, as well as *P. falciparum* + *P. ovale* may have implications on the training and developing competence of laboratory personnel in this setting to be able to report these species in the HMIS.

### Factors associated with asymptomatic malaria infection

Epidemiologically**,** malaria infection has been associated with factors related the vector, parasite, human host and the environment. However, in this study only the human host related factors were investigated. A number of studies have reported human behavioural and practices that are known to increase the risk of malaria transmission [20, 31]. In this study, the two factors found to be associated with asymptomatic malaria infection are sleeping under a mosquito net and presence of village health team or community health workers in community. Generally, individuals who reported having slept under mosquito nets were less likely to have asymptomatic malaria infection, aOR = 0.27 (95% CI 0.13–0.56), p = 0.001. The implication of this findings is that mosquito nets are providing an efficacious protective effect against malaria in this population. The observed effect of mosquito nets is consistent with several previous studies conducted elsewhere that have reported similar protective effect of mosquito nets against malaria [4]. The use of mosquito nets is one of the major WHO recommended interventions for malaria control [1, 43]. Mosquito nets are known to provide a physical barrier that protects individuals against mosquito bites. However, treated mosquito nets are also known to be impregnated with insecticides that kill the malaria vectors that rest on the nets before or after a blood meal. Consistent with this study, non-users of mosquito nets were at increased risk of carrying asymptomatic malaria infection in Ethiopian [27, 44] and Myanmar [28]. Similarly, individuals who reported presence of active village health teams/ community health worker in the community were less likely to have asymptomatic infection, aOR = 0.02 (95% CI 0.01–0.45), p = 0.001. Village health teams are resident in the village and are individuals usually without formal medical training who are equipped with skills for management and treatment of common illnesses such as malaria, pneumonia and diarrhoea at the village level. These groups are recognized by government and they are equipped with basic drugs such as anti-malarials, antibiotics, zinc and oral rehydration salts [4, 45]. The reduced odds of asymptomatic malaria in individuals where VHTs are functional suggests that the latter is an effective intervention that provides the first level of care for treatment and clearance of parasite reservoir from the community. In other studies elsewhere sex particularly being male was associated with asymptomatic infection than females (OR = 1.18, *p* = 0.015) [26], while in Kenya age was found to be a predictor [46].

### Performance of the diagnostic tests used

The study investigated the field performance of several diagnostic tools used for malaria testing during the survey in this setting. Accurate diagnosis of malaria parasites is important not only for administering correct treatment but also for surveillance and accurate estimation of disease burden to inform malaria control programme strategies. However, the diagnostic performance of RDTs can deteriorate and requires periodic monitoring [47–49]. In this study, expert blood smear microscopy was used as gold standard to evaluate the performance of two different RDTs. Study results showed that the P.f/pLDH RDTs had better sensitivity 90.0% (95% CI 86.5–93.5) and specificity 95.2% (95% CI 92.8–97.7%) compared to the routine HRP2 RDTs used by the MOH. Both the positive and negative predictive values were similarly higher for the *P. falciparum* pLDH RDTs. Although HRP2 RDTs are designed to detect the *P. falciparum* only, their low specificity (89%) could be attributed mainly due to their inability to detect the non-*P. falciparum* species observed in these samples [38] and similarly, its sensitivity (85%) is affected by its tendency to detect residual HRP2 antigen even after treatment and parasite clearance [50]. HRP2-only RDTs are currently the most commonly used tools for malaria diagnosis in Uganda and other parts of sub-Saharan Africa, where *P. falciparum* is the predominant parasite species [2, 43]. However, many factors can affect the effectiveness of RDTs as malaria diagnostic tools and require periodic monitoring [48, 49, 51, 52].

The decreased specificity of the HRP2 RDTs observed in this study suggests that HRP2-only RDTs can potentially miss detection of non-*P. falciparum* species reporting infected individuals as negative (false negative). This observation is consistent with the results of previous studies that showed the occurrence of false-negative HRP2 RDTs in non-*P. falciparum* clinical samples [42]. The presence of non-*P. falciparum* species in the study setting suggests that RDTs that target alternative antigens (other than HRP2 only) may be more appropriate for future use in case management and surveillance in this and other similar settings [53]. Previous RDT field studies in Uganda and elsewhere have reported comparable RDTs performance [54–57]. However, others studies reported contrary findings [40, 53, 58–61]. It is recognized that many other factors can affect the functionality of RDTs causing false-negative HRP2 RDTs in the field; these factors include product design, transport and storage conditions, parasite-related factors and operator-related factors [48, 62, 63]. Failure of the parasite to express the HRP2 target antigen or alterations in the HRP2 protein sequence has been shown to affect the efficacy of RDTs [34, 64, 65]. Variation in the pattern and sequence of histidine repeat tandems and the number, frequency and composition of amino acids within the HRP2 protein antigen are known to affect the efficacy of HRP2 RDTs [64–66].

Other known causes of false-negative RDTs include, transport and storage conditions, and user-related factors [48, 62]. However, in order to minimize user-related errors, the tests used in this study were quality-assured RDTs that are WHO prequalified and had passed the WHO product testing programme requirements [67, 68] and the users who performed the tests in the field were well-trained laboratory scientists.

## Limitations of the study

The study had limited geographical coverage and, therefore, generalizability may be not possible. Although blood smear microscopy was used as gold standard as recommended by the WHO [21, 43], the use of molecular tools such as PCR could have detected more asymptomatic infections.

## Conclusion

Asymptomatic malaria infections were present in the study population and this varied with place and person in the different age groups. *Plasmodium falciparum* was the dominant parasite species however the presence of *P. malariae* and *P. ovale* was observed which may have implication for the choice and deployment of diagnostic tools. Individuals sleeping under mosquito net and had presence of functional VHTs were less likely to have asymptomatic malaria infection. *Plasmodium falciparum* pLDH RDTs performed better than that the routinely used test HRP2 RDTs. In view of these findings, investigation and reporting of asymptomatic malaria reservoirs through community surveys is recommended for accurate disease burden estimate and better targeting of control interventions.

## Supplementary Information

**Additional file 1**. The detailed datasets analyzed are uploaded as additional data file 1.

## Data Availability

All data related to this study are fully available and have been uploaded as Additional file 1.

## Abbreviations

ACT: Artemisinin-based combination therapy
AIDS: Acquired immuno-deficiency Syndrome
DNA: Deoxyribonucleic acid
EIR: Entomological inoculation rate
Hb: Haemoglobin
HIV: Human immunodeficiency virus
HMIS: Health management information system
HRP2: Histidine rich protein 2
IPTp: Intermittent preventive treatment during pregnancy
LLINs: Long-lasting insecticidal nets
MOH: Ministry of health
NMCP: National malaria control programme
PCR: Polymerase chain reaction
RDT: Rapid diagnostic tests
UDHS: Uganda demographic and health survey
VHTs: Village health teams
WBCS: White blood cells
WHO: World Health Organization
