# Supplementary material for: Asymptomatic malaria infection, associated factors and accuracy of diagnostic tests in a historically high transmission setting in Northern Uganda
Source: Malar J. 2022 Dec 23;21:392. doi: 10.1186/s12936-022-04421-1 (PMC9783970; doi:10.1186/s12936-022-04421-1)
Supplement: Supplementary file 1 — Additional file 1. The detailed datasets analyzed are uploaded as additional data file 1. [file 12936_2022_4421_MOESM1_ESM.pdf]

ID	SEX	VILLAGE	Age	Knowledge and recognition of malaria symptoms	is there a CW or VHT member who distributes medicines in your village or community	Did you sleep under a mosquito net the previous night?	Blood Smear
1	female	AKWON	3	fever	no	No	Positive
2	female	AKWON	3	fever	no	No	Positive
3	male	AKWON	3	fever	yes	Yes	Negative
4	female	AKWON	3	fever	yes	No	Negative
5	female	AKWON	2	fever	no	No	Positive
6	male	AKWON	2	fever	no	Yes	Positive
7	female	AKWON	2	fever	yes	Yes	Negative
8	male	AKWON	3	fever			Negative
9	male	AKWON	3	fever	yes	No	Positive
10	female	AKWON	3	other	yes	No	Positive
11	female	AKWON	3	fever	yes	No	Negative
12	female	AKWON	3	fever	yes	Yes	Negative
13	male	AKWON	3	fever	yes	Yes	Negative
14	female	AKWON	3	other	yes	Yes	Negative
15	male	AKWON	3	fever	yes	Yes	Negative
16	male	AKWON	3	fever	yes	Yes	Positive
17	male	AKWON	3	fever	yes	Yes	Positive
18	female	AKWON	3	fever	yes	Yes	Negative
19	male	AKWON	2	other	no	Yes	Positive
20	female	AKWON	3	fever	yes	No	Negative
21	male	AKWON	3	fever	no	Yes	Positive
22	female	AKWON	3	fever	yes	Yes	Negative
23	male	AKWON	3	fever	yes	Yes	Negative
24	male	AKWON	3	other	no	No	Positive
25	male	AKWON	3	other	yes	Yes	Negative
26	female	AKWON	2	fever	yes	No	Positive
27	male	AKWON	2	fever	no	Yes	Negative
28	female	AKWON	3	fever	no	No	Positive
29	female	AKWON	2	fever	no	No	Positive
30	female	AKWON	3	fever	no	No	Negative
31	male	AKWON	3	fever	yes	No	Positive
32	female	AKWON	3	fever	yes	Yes	Negative
33	male	AKWON	3	fever	yes	Yes	Negative
34	female	AKWON	3	fever	yes	Yes	Negative
35	female	AKWON	3	other	yes	Yes	Negative
36	female	AKWON	3	fever	yes	Yes	Negative
37	male	AKWON	3	fever	yes	No	Negative
38	female	AKWON	3	fever	yes	Yes	Negative
39	male	AKWON	3	fever	yes	Yes	Negative
40	female	AKWON	3	fever	yes	Yes	Negative
41	male	AKWON	3	other	no	No	Positive
42	female	AKWON	3	fever	no	No	Positive
43	male	AKWON	2	fever	yes	Yes	Negative
44	female	AKWON	1	fever	yes	No	Negative
45	female	AKWON	1	fever	yes	No	Negative
46	female	AKWON	1	fever	yes	Yes	Negative
47	male	AKWON	1	fever	no	No	Positive
48	female	AKWON	2	other	yes	Yes	Negative
49	female	AKWON	2	other	yes	No	Positive
50	female	AKWON	3	other	yes	Yes	Negative
51	male	AKWON	2	fever	yes	Yes	Negative
52	male	AKWON	2	fever	yes	Yes	Negative

53	male	AKWON	1	fever	yes	Yes	Negative
54	female	AKWON	1	fever	yes	Yes	Negative
55	male	AKWON	1	other	yes	No	Negative
56	female	AKWON	1	fever	yes	Yes	Negative
57	female	AKWON	2	fever	yes	No	Positive
58	male	AKWON	2	fever	no	No	Negative
59	male	AKWON	3	fever	yes	Yes	Negative
60	female	AKWON	3	fever	yes	Yes	Negative
61	male	AKWON	3	fever	yes	No	Negative
62	female	AKWON	3	fever	yes	No	Positive
63	female	AKWON	3	fever	yes	Yes	Negative
64	female	AKWON	3	fever	yes	Yes	Negative
65	male	AKWON	3	fever	yes	Yes	Negative
66	male	AKWON	3	fever	yes	Yes	Positive
67	male	AKWON	3	fever	yes	Yes	Negative
68	male	AKWON	3	fever	yes	Yes	Negative
69	female	AKWON	3	fever	yes	No	Negative
70	male	AKWON	1	fever	yes	Yes	Negative
71	male	EGUM	1	fever	yes	Yes	Negative
72	female	EGUM	1	fever	no	No	Positive
73	female	EGUM	2	other	yes	Yes	Negative
74	female	EGUM	2	other	yes	Yes	Negative
75	female	EGUM	2	fever	yes	Yes	Negative
76	female	EGUM	2	other	yes	No	Negative
77	female	EGUM	2	other	yes	No	Negative
78	female	EGUM	1	fever	no	No	Positive
79	female	EGUM	1	fever	yes	Yes	Negative
80	male	EGUM	2	fever	no	Yes	Positive
81	male	EGUM	2	fever	no	No	Positive
82	male	EGUM	2	fever	yes	Yes	Negative
83	male	EGUM	3	fever	yes	Yes	Negative
84	female	EGUM	3	fever	no	No	Positive
85	male	EGUM	3	fever	no	No	Positive
86	male	EGUM	3	fever	yes	Yes	Negative
87	female	EGUM	3	fever	no	Yes	Positive
88	male	EGUM	3	fever	yes	No	Negative
89	female	EGUM	2	fever	yes	Yes	Negative
90	female	EGUM	2	fever	yes	Yes	Negative
91	female	EGUM	2	fever	no	No	Positive
92	female	EGUM	2	fever	yes	Yes	Negative
93	male	EGUM	1	fever	yes	Yes	Negative
94	female	EGUM	1	fever	yes	Yes	Negative
95	female	EGUM	1	fever	yes	Yes	Negative
96	male	EGUM	3	fever	yes	Yes	Negative
97	male	EGUM	3	fever	yes	Yes	Negative
98	male	EGUM	3	fever	yes	Yes	Negative
99	female	EGUM	1	fever	yes	Yes	Negative
100	male	EGUM	1	fever	yes	Yes	Negative
101	male	EGUM	1	fever	yes	Yes	Negative
102	female	EGUM	1	fever	no	No	Positive
103	female	EGUM	3	fever	no	No	Positive
104	male	EGUM	3	fever	yes	Yes	Negative
105	male	EGUM	3	fever	yes	No	Negative
106	female	EGUM	2	fever	no	No	Positive
107	female	EGUM	2	fever	no	No	Positive
108	female	EGUM	2	fever	yes	Yes	Negative
109	female	EGUM	1	fever	yes	No	Negative
110	male	EGUM	1	fever	yes	No	Negative

111	male	EGUM	1	fever	yes	Yes	Negative
112	female	EGUM	1	fever	yes	Yes	Negative
113	female	EGUM	3	fever	yes	No	Negative
114	male	EGUM	3	fever	yes	No	Negative
115	female	EGUM	3	fever	yes	Yes	Negative
116	male	EGUM	3	fever	yes	Yes	Negative
117	female	EGUM	3	fever	yes	Yes	Negative
118	male	EGUM	3	fever	yes	Yes	Negative
119	male	EGUM	3	fever	yes	Yes	Negative
120	male	EGUM	2	fever	yes		Positive
121	male	EGUM	2	fever	yes	No	Negative
122	female	EGUM	2	fever	no	Yes	Positive
123	female	EGUM	2	fever	no	Yes	Positive
124	male	EGUM	3	fever	yes	Yes	Negative
125	female	EGUM	3	fever	yes	No	Negative
126	male	EGUM	3	fever	yes	Yes	Negative
127	male	EGUM	3	fever	yes	No	Positive
128	male	EGUM	2	fever	yes	No	Positive
129	male	EGUM	2	fever	yes	No	Negative
130	male	EGUM	2	fever	yes	No	Positive
131	female	EGUM	2	fever	yes	No	Positive
132	male	EGUM	3	fever	yes	No	Positive
133	male	EGUM	1	fever	yes	Yes	Negative
134	female	EGUM	1	fever	yes	No	Negative
135	female	EGUM	1	fever	yes	No	Positive
136	female	EGUM	2	fever	yes	Yes	Negative
137	male	EGUM	2	fever	yes	Yes	Negative
138	female	EGUM	3	fever	yes	Yes	Negative
139	male	EGUM	2	fever	no	No	Positive
140	male	EGUM	3	fever	no	Yes	Positive
141	male	GWERI	3	fever	yes	Yes	Negative
142	male	GWERI	2	fever	yes	No	Positive
143	male	GWERI	2	fever	yes	Yes	Negative
144	female	GWERI	2	fever	yes	Yes	Negative
145	female	GWERI	1	fever	yes	Yes	Negative
146	male	GWERI	1	fever	yes	Yes	Negative
147	male	GWERI	1	fever	no	No	Positive
148	female	GWERI	3	fever	no	No	Positive
149	female	GWERI	3	fever	yes	Yes	Negative
150	female	GWERI	3	fever	yes	Yes	Negative
151	male	GWERI	1	fever	yes	Yes	Negative
152	male	GWERI	2	fever	yes	Yes	Negative
153	male	GWERI	1	fever	no	Yes	Positive
154	male	GWERI	3	fever	yes	No	Negative
155	male	GWERI	3	fever	yes	Yes	Negative
156	female	GWERI	1	fever	yes	Yes	Negative
157	female	GWERI	3	fever	no	No	Positive
158	female	GWERI	3	fever	yes	No	Negative
159	female	GWERI	2	fever	yes	Yes	Negative
160	female	GWERI	2	fever	yes	No	Negative
161	male	GWERI	3	fever	no	No	Positive
162	female	GWERI	1	fever	no	No	Positive
163	male	GWERI	2	fever	no	No	Positive
164	male	GWERI	1	fever	yes	Yes	Negative
165	female	GWERI	3	fever	yes	No	Negative
166	male	GWERI	3	fever	yes	Yes	Negative
167	male	GWERI	3	fever	yes	Yes	Negative
168	male	GWERI	2	fever	no	no	Positive

169	male	GWERI	1	fever	yes	Yes	Negative
170	male	GWERI	3	fever	yes	Yes	Negative
171	female	GWERI	2	fever	no	Yes	Positive
172	female	GWERI	2	fever	yes	No	Negative
173	male	GWERI	1	fever	no	No	Positive
174	female	GWERI	3	fever	yes	No	Negative
175	male	GWERI	2	fever	no	No	Positive
176	female	GWERI	2	fever	yes	Yes	Negative
177	female	GWERI	1	fever	yes	Yes	Negative
178	female	GWERI	3	fever	no	No	Positive
179	female	GWERI	1	fever	yes	Yes	Negative
180	male	GWERI	2	fever	yes	No	Negative
181	male	GWERI	2	fever	no	No	Positive
182	male	GWERI	3	fever	no	Yes	Positive
183	male	GWERI	3	fever	yes	Yes	Negative
184	female	GWERI	3	fever	yes	Yes	Negative
185	male	GWERI	3	other	yes	Yes	Negative
186	male	GWERI	1	fever	yes	Yes	Negative
187	male	GWERI	1	fever	yes	Yes	Negative
188	female	GWERI	2	fever	no	No	Positive
189	male	GWERI	1	fever	no	Yes	Positive
190	female	GWERI	3	fever	no	No	Negative
191	male	GWERI	1	fever	yes	Yes	Negative
192	male	GWERI	1	fever	no	No	Positive
193	female	GWERI	1	fever	yes	Yes	Negative
194	female	GWERI	1	fever	yes	No	Negative
195	female	GWERI	1	fever	yes	No	Negative
196	female	GWERI	1	fever	no	No	Positive
197	female	GWERI	3	fever	yes	Yes	Negative
198	female	GWERI	1	fever	yes	Yes	Positive
199	female	GWERI	3	fever	yes	Yes	Negative
200	female	GWERI	1	fever	yes	No	Positive
201	male	GWERI	1	fever	no	Yes	Positive
202	male	GWERI	1	fever	yes	Yes	Negative
203	female	GWERI	3	fever	no	Yes	Positive
204	female	GWERI	2	fever	no	No	Positive
205	female	GWERI	2	fever	yes	Yes	Negative
206	female	GWERI	2	fever	yes	No	Positive
207	female	GWERI	3	fever	yes	No	Positive
208	female	GWERI	3	fever	no	Yes	Positive
209	male	GWERI	1	fever	yes	Yes	Negative
211	female	AMIA A	3	fever	yes	Yes	Negative
211	male	AMIA A	3	fever	yes	Yes	Negative
212	female	AMIA A	3	other	yes	Yes	Positive
213	female	AMIA A	1	fever	yes	Yes	Negative
214	female	AMIA A	2	other	yes	Yes	Positive
215	female	AMIA A	2	other	yes	Yes	Negative
216	female	AMIA A	1	fever	yes	Yes	Negative
217	female	AMIA A	2	fever	yes	Yes	Negative
218	male	AMIA A	3	fever	yes	Yes	Negative
219	female	AMIA A	2	fever	yes	Yes	Negative
220	male	AMIA A	2	fever	yes	Yes	Positive
221	female	AMIA A	1	fever	yes	No	Positive
222	female	AMIA A	2	fever	yes	No	Negative
223	female	AMIA A	3	fever	yes	Yes	Negative
224	female	AMIA A	3	other	yes	No	Negative
225	male	AMIA A	2	fever	yes	Yes	Negative
226	female	AMIA A	1	fever	yes	Yes	Negative

227	male	AMIA A	2	fever	yes	Yes	Negative
228	female	AMIA A	2	fever	yes	No	Negative
229	female	AMIA A	2	fever	yes	No	Positive
230	male	AMIA A	3	fever	yes	Yes	Negative
231	male	AMIA A	2	fever	yes	Yes	Negative
232	female	AMIA A	3	fever	no	Yes	Positive
233	female	AMIA A	3	fever	yes	Yes	Negative
234	female	AMIA A	1	fever	no	Yes	Positive
235	female	AMIA A	3	fever	yes	No	Negative
236	female	AMIA A	3	fever	yes	No	Negative
237	male	AMIA A	1	fever	no	No	Positive
238	male	AMIA A	1	fever	yes	Yes	Negative
239	male	AMIA A	1	fever	yes	Yes	Negative
240	male	AMIA A	2	fever	no	No	Positive
241	male	AMIA A	1	fever	no	No	Positive
242	male	AMIA A	2	fever	yes	No	Negative
243	female	AMIA A	3	fever	yes	No	Negative
244	female	AMIA A	1	fever	yes	Yes	Positive
245	male	AMIA A	1	fever	yes	Yes	Positive
245	male	AMIA A	2	fever	yes	Yes	Negative
247	male	AMIA A	3	fever	no	Yes	Positive
248	male	AMIA A	1	fever	no	Yes	Positive
249	female	AMIA A	1	other	no	No	Negative
250	male	AMIA A	3	fever	yes	No	Negative
251	male	AMIA A	3	fever	yes	No	Positive
252	female	AMIA A	3	fever			Positive
253	female	AMIA A	1	fever	yes	No	Negative
254	female	AMIA A	2	fever	yes	No	Negative
255	female	AMIA A	2	fever	no	No	Positive
256	male	AMIA A	2	fever	no	Yes	Positive
257	female	AMIA A	2	fever			Positive
258	male	AMIA A	2	fever	yes	No	Negative
259	female	AMIA A	2	fever	yes	No	Negative
260	female	AMIA A	2	fever	yes	No	Negative
261	female	AMIA A	1	other	yes	No	Negative
262	female	AMIA A	1	fever	yes	No	Negative
263	female	AMIA A	1	other	yes	No	Negative
264	male	AMIA A	2	other	yes	No	Positive
265	female	AMIA A	1	other	yes	Yes	Negative
266	male	AMIA A	3	fever	yes	No	Positive
267	male	AMIA A	2	other	yes	no	Negative
268	male	AMIA A	1	fever	yes	No	Negative
269	male	AMIA A	1	fever	yes	No	Negative
270	male	AMIA A	1	fever	yes	Yes	Negative
271	female	AMIA A	1	fever	yes	Yes	Negative
272	female	AMIA A	1	fever	yes	Yes	Negative
273	male	AMIA A	1	fever	yes	Yes	Negative
274	female	AMIA A	2	fever	no	Yes	Positive
275	female	AMIA A	3	fever	no	Yes	Positive
276	female	AMIA A	1	fever	no	no	Positive
277	female	AMIA A	2	fever	no	no	Positive
278	female	AMIA A	1	other	yes	no	Negative
279	female	AMIA A	1	other	yes	Yes	Negative
280	male	AMIA A	2	other	yes	Yes	Positive
281	male	AMIA A	3	fever	yes	Yes	Negative
282	male	AMIA A	2	fever	yes	Yes	Negative
283	male	AMIA A	3	other	yes	Yes	Negative
284	female	AMIA A	2	fever	yes	Yes	Positive

285	female	AMIA A	1	fever	yes	Yes	Negative
286	male	AMIA A	1	fever	yes	Yes	Negative
287	male	AMIA A	1	other	yes	Yes	Negative
288	male	AMIA A	1	other	yes	Yes	Positive
